# Global, regional, and national burden of cancers attributable to occupational risks from 1990 to 2019

**DOI:** 10.1093/joccuh/uiae040

**Published:** 2024-07-24

**Authors:** Shiliang Ling, Lihong Zhou, Yanfeng Wu, Xiaoling Zhang, Wulong Han, Lihua Cui, Zhiyu Luan

**Affiliations:** Department of Oncology, Ningbo Municipal Hospital of Traditional Chinese Medicine (TCM), Affiliated Hospital of Zhejiang Chinese Medical University, Ningbo, Zhejiang, China; Department of Spleen and Stomach, Shanghai Pudong New Area Traditional Chinese Medicine Hospital, Shanghai, China; Department of Anesthesiology, Ningbo Municipal Hospital of Traditional Chinese Medicine (TCM), Affiliated Hospital of Zhejiang Chinese Medical University, Ningbo, Zhejiang, China; Department of Oncology, Ningbo Municipal Hospital of Traditional Chinese Medicine (TCM), Affiliated Hospital of Zhejiang Chinese Medical University, Ningbo, Zhejiang, China; Department of Oncology, Ningbo Municipal Hospital of Traditional Chinese Medicine (TCM), Affiliated Hospital of Zhejiang Chinese Medical University, Ningbo, Zhejiang, China; Department of Oncology, Ningbo Municipal Hospital of Traditional Chinese Medicine (TCM), Affiliated Hospital of Zhejiang Chinese Medical University, Ningbo, Zhejiang, China; Department of Oncology, Ningbo Municipal Hospital of Traditional Chinese Medicine (TCM), Affiliated Hospital of Zhejiang Chinese Medical University, Ningbo, Zhejiang, China

**Keywords:** cancer, occupational risk factors, death, disability-adjusted life year, estimated annual percentage change

## Abstract

**Background:**

Based on data from the Global Burden of Disease study, the burden of cancer attributable to occupational risks between 1990 and 2019 was explored.

**Methods:**

The estimated burden in different regions was compared in terms of the age-standardized death rates (ASDRs), age-standardized disability-adjusted life years (DALYs) rates, and corresponding estimated annual percentage changes (EAPCs). The comparative risk assessment framework was used to estimate the risk of death and DALYs attributable to occupational risk factors.

**Results:**

Globally from 1990 to 2019, ASDRs decreased (EAPC = −0.69; 95% CI: −0.76 to −0.61), and age-standardized DALY rates decreased (EAPC = −0.99; 95% CI: −1.05 to −0.94). In terms of the global age distribution of cancer attributable to occupational risk factors, the death rate and DALY rates increased with age. In addition, from 1990 to 2019, the number of deaths, DALYs, ASDRs, and age-standardized DALY rates in men were higher than those in women, and the cancer burden grew fastest in Georgia (EAPC = 5.04), Croatia (EAPC = 4.01), and Honduras (EAPC = 3.54). Moreover, as the sociodemographic index (SDI) value of a country or region increased, its burden of cancer attributable to occupational risk factors rapidly increased.

**Conclusions:**

The global cancer burden attributable to occupational risk factors declined from 1990 to 2019, was higher in men than in women, and was concentrated in middle-aged and older adults. The baseline cancer burdens of regions or countries increased as their SDI values increased and were especially high in high-SDI regions or countries.

## Key points

**What is already known on this topic:** 

Prior to this study, the scientific knowledge about the global burden of cancers attributable to occupational risks was limited, especially at a global and national level. Few studies had assessed and compared cancer burdens related to occupational risk factors comprehensively.

**What this study adds:** 

This study provides a comprehensive analysis of the global burden of cancers attributable to occupational risks from 1990 to 2019. The study shows that the burden was higher in men than in women, and concentrated in middle-aged and older adults. Additionally, as the SDI value of a region increased, its cancer burden attributable to occupational risks also increased, particularly in high-SDI regions.

**How this study might affect research, practice, or policy:** 

The findings from this study could significantly influence future research directions by highlighting the need for targeted cancer prevention strategies in different demographic and socioeconomic groups. For health care practice, the study underscores the importance of awareness and preventive measures in occupational settings to mitigate cancer risks. The data can aid in prioritizing health care resources and interventions in regions with the highest burdens.

## 1. Introduction

Cancer is a major global public health challenge. In 2020, there were over 19 million new cancer cases and over 10 million cancer deaths worldwide, which represents an enormous disease burden.^1^^,^^2^ Therefore, studying the pathogenesis of cancer and its risk factors, and identifying effective prevention and treatment methods, are crucial aspects of global public health efforts.

Occupational risks factors for cancer may be a key cause of high cancer incidence and death rates. Moreover, these risk factors, which include carcinogen exposure, radiation exposure, occupational infection, and occupational pneumoconiosis, are preventable. In recent years, an increasing number of studies have found a close relationship between occupational risk factors and the occurrence of cancer.^3^ For example, Scelo G et al found that some chemicals in occupational environments, such as arsenic and certain organic solvents, may increase the risk of kidney cancer.^4^ Moreover, Burger M et al found that occupational exposure to solvents, dust, and various chemicals may increase the risk of bladder cancer,^5^ and that these exposures may be more common in certain occupations than in others. Therefore, people working in these occupations may have a higher risk of cancer.^3–5^

However, few studies have assessed and compared cancer burdens related to occupational risk factors at the global and national levels.^6–9^ Therefore, it is crucial to understand the global burden of cancer and whether its change trends are attributable to occupational risk factors, as this will support determination of global priorities for cancer prevention. Accordingly, we analyzed data from the Global Burden of Disease (GBD) study 2019 to determine the global burden of cancer attributable to occupational risk factors from 1990 to 2019; to identify the change trend of age-standardized death rates (ASDRs) and age-standardized disability-adjusted life year (DALY) rate of cancer attributable to occupational risk factors; and to examine how this burden was affected by various factors, such as age, gender, and sociodemographic index (SDI). Our results enhance the understanding of global trends in cancer burdens attributable to occupational risk factors, and provide strong support for public health decision-making.^6^

## 2. Methods

### 2.1. Research data

The GBD 2019 included literature, surveys, and epidemiological data collected and sorted by more than 3600 researchers from more than 145 countries. Specifically, it calculated and analyzed epidemiological data on more than 350 diseases in 204 countries and regions, including incidence rate, prevalence, death rate, and DALY data.

The current study used the GBD 2019 database to determine the cancer ASDRs and the age-standardized DALY rates and corresponding estimated annual percentage changes (EAPCs) attributable to occupational risk factors from 1990 to 2019; and to analyze the global cancer burden attributable to these risk factors and its change trend.

The SDI is a composite indicator of the extent of development of a country or region.^8^ An SDI value of 0 represents the minimum level of each covariate input and a condition in which the selected health outputs are absent, whereas an SDI value of 1 represents the maximum level of each covariate input and a condition in which the selected health outputs cannot increase further.^10^ The 11 geographic regions were categorized into 5 groups according to their SDI (low, low-middle, middle, high-middle, and high SDI).

The Bayesian meta-regression modeling tool DisMod-MR 2.1 was used to analyze data and determine 95% uncertainty intervals (UIs).^9^^,^^11^ This study was reviewed and approved by Ningbo Traditional Chinese Medicine Hospital.^12^

### 2.2. Case definition of cancer

Diagnoses of cancer in GBD 2019 were made according to the International Classification of Diseases (ICD)-9 codes 151-151.9, 211.1, and 230.2, and ICD-10 codes C16-16.9, D00.2, D13.1, and D37.1.

### 2.3. Definition of occupational risk factors

Occupational risks included occupational carcinogens, occupational exposure to asbestos, arsenic, benzene, beryllium, cadmium, chromium, diesel engine exhaust, formaldehyde, nickel, polycyclic aromatic hydrocarbons, silica, sulfuric acid, and trichloroethylene, as well as occupational asthmagens, particulate matter, gases, and fumes, noise, injuries, and ergonomic factors.^13–15^

### 2.4. Risk factor analysis

The GBD adopts the comparative risk assessment (CRA) framework, which is a comprehensive method for quantifying risk factors and a useful tool for determining the correlation between a risk factor and its outcomes. Every year, researchers update the CRA of the GBD to incorporate improved methods, new risk and risk–outcome pairings, and new data on risk-exposure levels and risk–outcome correlations.^16^ Accordingly, we used the CRA framework to estimate the levels and trends of exposure to occupational risk factors for cancer, and deaths and DALYs attributable to these risk factors, for each age group, gender, year, and location. In addition, we employed the counterfactual scenario of theoretical minimum risk-exposure level to estimate the proportion of deaths and DALYs attributable to a given risk factor.

### 2.5. Statistical analysis

We calculated age-standardized rates and described the trends in their changes in terms of EAPCs. We regarded cases in which the lower limit of the EAPC and its 95% CI were both greater than 0 as representing decreasing age-standardized rates, and those in which the upper limit of the EAPC and its 95% CI were both less than 0 as representing decreasing age-standardized rates. If a case exhibited neither of these patterns, we considered the change in its age-standardized rate to be relatively stable (ie, not changing significantly).^11^ The relationships between the EAPCs and age-standardized rates, and between the SDI values and EAPCs, were calculating by Pearson’s correlation coefficients (ρ). Spatiotemporal Gaussian process regression has been utilized in previous studies of GBD to estimate exposure to multiple risks, particularly for risks with detailed age- and sex-specific data. This method leverages information across space, time, and age to accurately estimate the underlying trend of a particular risk. With adequate data, spatiotemporal Gaussian process regression proves to be a rapid and adaptable modeling approach that is well suited for capturing nonlinear time trends.^17^^,^^18^

All statistical analyses were conducted using R software (version 4.1.3; R Foundation). A 2-sided *P*-value of less than .05 was considered statistically significant.

## 3. Results

### 3.1. Trends in the global burden of cancer from 1990 to 2019 attributable to occupational risk factors

**Figure 1:**
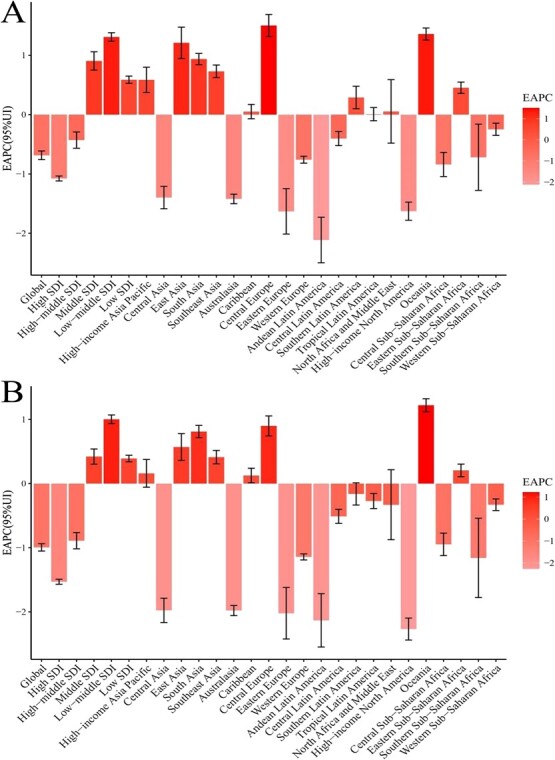
The estimated annual percentage change (EAPC) of age-standardized rates in cancer attributed to occupational risks from 1990 to 2019, by region. A, The EAPC of age standardized death rate. B, The EAPC of age-standardized disability-adjusted life year (DALY) rate.

From 1990 to 2019, the ASDR for cancer decreased from 5.17 to 4.17; and the age-standardized DALY rate due to cancer decreased from 112.44 to 84.42 (Table 1, Figure 1). Moreover, from 1990 to 2019, the EAPC in the ASDR for cancer was −0.69 (95% CI: −0.76 to −0.61), and the EAPC of the age-standardized DALY rate due to cancer was −0.99 (95% CI: −1.05 to −0.94) (Table 1, Figure 1).

**Table 1:** The numbers and age-standardized rates of cancer attributed to occupational risks from 1990 to 2019, by region.

**Characteristics**	**1990**				**2019**				**EAPC (1990-2019)**	
	**Death cases**	**Age-standardized death rate per 10 000**	**DALYs**	**Age-standardized DALY rate per 10 000**	**Death cases**	**Age-standardized death rate per 10 000**	**DALYs**	**Age-standardized DALY rate per 10 000**	**Age-standardized death rate**	**Age-standardized DALY rate**
	**No. (95% UI)**	**No. (95% UI)**	**No. (95% UI)**	**No. (95% UI)**	**No. (95% UI)**	**No. (95% UI)**	**No. (95% UI)**	**No. (95% UI)**	**No. (95% UI)**	**No. (95% UI)**
**Global**	195422.60 (153511.04 to 236508.67)	5.17 (4.08 to 6.25)	4525449.51 (3573000.79 to 5519304.47)	112.44 (88.83 to 136.89)	333867.01 (263491.40 to 404640.86)	4.17 (3.29 to 5.05)	6964775.16 (5467884.04 to 8580431.22)	84.42 (66.24 to 103.71)	−0.69 (−0.76 to −0.61)	−0.99 (−1.05 to −0.94)
**Sex**										
**Female**	31820.69 (23915.72 to 40210.20)	1.52 (1.14 to 1.92)	765154.11 (568943.65 to 960946.85)	35.24 (26.15 to 44.36)	66480.37 (48651.08 to 84957.74)	1.52 (1.11 to 1.94)	1441729.30 (1067075.99 to 1829687.11)	33.10 (24.50 to 42.01)	−0.03 (−0.10 to 0.03)	−0.33 (−0.38 to −0.27)
**Male**	163601.91 (126895.37 to 202606.48)	10.08 (7.76 to 12.49)	3760295.41 (2900772.24 to 4643567.86)	206.29 (159.87 to 254.45)	267386.64 (206130.40 to 330673.03)	7.54 (5.78 to 9.34)	5523045.87 (4272769.09 to 6829138.09)	144.38 (111.96 to 178.45)	−0.90 (−0.99 to −0.82)	−1.20 (−1.26 to −1.13)
**Sociodemographic index (SDI)**										
**Low SDI**	2344.35 (1535.24 to 4158.42)	0.98 (0.64 to 1.88)	68779.86 (45260.02 to 111082.16)	25.65 (16.77 to 44.21)	5885.12 (4140.95 to 9376.79)	1.17 (0.81 to 1.96)	165161.79 (117115.74 to 246524.91)	28.83 (20.30 to 44.87)	0.59 (0.53 to 0.65)	0.39 (0.34 to 0.44)
**Low-middle SDI**	7459.75 (5575.32 to 9606.88)	1.24 (0.92 to 1.64)	219569.39 (161634.21 to 279696.90)	32.51 (24.22 to 41.46)	22999.10 (17798.98 to 29001.16)	1.73 (1.33 to 2.17)	604472.67 (465727.83 to 762733.48)	41.91 (32.38 to 52.66)	1.31 (1.24 to 1.38)	1.00 (0.94 to 1.07)
**Middle SDI**	21662.28 (16228.92 to 27330.63)	2.09 (1.59 to 2.62)	635370.46 (474809.71 to 805643.83)	55.00 (41.08 to 69.53)	62766.33 (46998.25 to 81790.94)	2.57 (1.93 to 3.30)	1585989.94 (1175171.87 to 2062854.49)	60.42 (45.15 to 78.84)	0.91 (0.75 to 1.06)	0.42 (0.30 to 0.54)
**High-middle SDI**	51269.06 (39416.00 to 63746.76)	4.78 (3.68 to 5.92)	1307842.99 (997209.77 to 1639173.46)	117.48 (90.12 to 147.57)	86011.72 (66405.69 to 107788.46)	4.19 (3.24 to 5.25)	1905633.96 (1476519.22 to 2394184.61)	92.62 (71.82 to 116.22)	−0.43 (−0.57 to −0.29)	−0.89 (−1.02 to −0.76)
**High SDI**	112592.47 (88018.79 to 135694.87)	10.46 (8.19 to 12.62)	2291735.75 (1791631.82 to 2779091.69)	217.80 (170.97 to 264.63)	156043.35 (123033.13 to 189065.06)	7.67 (6.08 to 9.32)	2700006.71 (2137968.42 to 3298446.45)	141.99 (112.11 to 173.96)	−1.08 (−1.12 to −1.03)	−1.53 (−1.57 to −1.49)
**Region**										
**Andean Latin America**	562.47 (380.68 to 742.36)	2.88 (1.94 to 3.83)	13895.68 (9724.90 to 18225.06)	65.20 (44.87 to 85.89)	977.82 (676.96 to 1329.02)	1.77 (1.23 to 2.42)	22836.25 (16017.07 to 31277.01)	39.95 (27.86 to 54.55)	−2.11 (−2.50 to −1.73)	−2.13 (−2.55 to −1.72)
**Australasia**	3583.56 (2906.76 to 4229.31)	14.79 (11.98 to 17.45)	74618.04 (60351.03 to 88404.65)	310.85 (249.89 to 368.24)	5270.17 (4262.76 to 6185.70)	9.97 (8.11 to 11.72)	91710.25 (74396.32 to 108316.25)	181.71 (148.14 to 215.55)	−1.42 (−1.50 to −1.34)	−1.98 (−2.06 to −1.90)
**Caribbean**	578.94 (445.44 to 728.40)	2.26 (1.73 to 2.85)	14355.62 (11035.87 to 17917.96)	54.05 (41.59 to 67.55)	1189.49 (862.81 to 1592.70)	2.29 (1.66 to 3.07)	29289.37 (21381.24 to 39033.44)	56.20 (41.03 to 74.83)	0.05(−0.07 to 0.17)	0.13 (0.01 to 0.24)
**Central Asia**	1722.14 (1287.762 to 198.90)	3.42 (2.57 to 4.34)	52862.86 (39033.25 to 67392.41)	101.39 (75.79 to 129.45)	1866.66 (1416.58 to 2335.62)	2.47 (1.88 to 3.11)	52878.48 (40672.85 to 66002.17)	63.33 (48.38 to 79.05)	−1.40 (−1.59 to −1.21)	−1.98 (−2.17 to −1.79)
**Central Europe**	5621.73 (3827.65 to 7497.30)	3.69 (2.51 to 4.93)	153836.53 (102185.82 to 208105.10)	101.31 (67.27 to 137.00)	10912.15 (7600.13 to 14763.95)	5.02 (3.51 to 6.81)	248985.40 (174051.36 to 341120.50)	119.86 (83.16 to 164.34)	1.50 (1.32 to 1.69)	0.90 (0.74 to 1.05)
**Central Latin America**	1522.18 (1223.82 to 1846.40)	1.85 (1.48 to 2.26)	41228.29 (33043.21 to 50096.68)	45.56 (36.50 to 55.40)	3942.07 (3028.92 to 5066.95)	1.68 (1.30 to 2.17)	97738.80 (74955.07 to 127211.20)	40.47 (31.10 to 52.57)	−0.40 (−0.52 to −0.29)	−0.51 (−0.62 to −0.40)
**Central sub-Saharan Africa**	406.11 (205.64 to 874.29)	1.78 (0.89 to 4.28)	11964.63 (6277.55 to 23879.17)	45.90 (23.44 to 98.61)	777.22 (401.50 to 1687.12)	1.49 (0.73 to 3.59)	22754.82 (11996.80 to 46331.96)	37.18 (19.25 to 80.22)	−0.84 (−1.04 to −0.64)	−0.95 (−1.12 to −0.77)
**East Asia**	23579.34 (16701.56 to 31194.08)	2.64 (1.90 to 3.48)	688870.21 (483821.23 to 917913.90)	70.35 (49.83 to 93.45)	69444.87 (49531.40 to 92102.70)	3.36 (2.39 to 4.49)	1694944.88 (1196210.55 to 2281032.26)	78.15 (55.35 to 104.29)	1.21 (0.95 to 1.48)	0.57 (0.36 to 0.78)
**Eastern Europe**	9696.73 (6767.27 to 12804.47)	3.32 (2.30 to 4.34)	271466.58 (183887.00 to 359249.76)	92.96 (63.28 to 123.10)	8734.95 (6201.00 to 11578.74)	2.51 (1.79 to 3.33)	217817.50 (151742.30 to 288469.85)	64.38 (44.93 to 85.13)	−1.63 (−2.01 to −1.25)	−2.02 (−2.42 to −1.62)
**Eastern sub-Saharan Africa**	745.97 (439.05 to 1869.06)	1.01 (0.56 to 2.71)	21574.58 (13267.78 to 47715.49)	25.45 (15.17 to 61.34)	1826.76 (1099.97 to 4249.89)	1.18 (0.67 to 2.92)	50783.39 (32589.06 to 106871.98)	27.96 (16.98 to 63.59)	0.45 (0.36 to 0.55)	0.21 (0.11 to 0.30)
**High-income Asia Pacific**	8716.20 (6520.03 to 11048.52)	4.51 (3.39 to 5.71)	175718.96 (128501.27 to 224775.20)	86.75 (63.74 to 110.77)	25331.30 (18040.23 to 32833.78)	4.78 (3.45 to 6.17)	384218.98 (278741.87 to 494947.18)	82.54 (60.23105.61)	0.59 (0.38 to 0.80)	0.16(−0.06 to 0.38)
**High-income North America**	42766.93 (32789.04 to 52260.31)	11.64 (8.93 to 14.23)	853026.63 (654583.63 to 1050250.03)	240.93 (184.29 to 299.49)	52743.04 (41097.45 to 64733.64)	7.99 (6.22 to 9.79)	906160.11 (698139.12 to 1126183.10)	141.32 (109.20175.37)	−1.63 (−1.78 to −1.47)	−2.27 (−2.44 to −2.10)
**North Africa and Middle East**	5386.50 (3785.30 to 7262.24)	3.16 (2.25 to 4.24)	148241.86 (104488.66 to 201325.26)	79.25 (56.05 to 106.91)	11326.03 (8077.01 to 15503.06)	2.75 (1.95 to 3.79)	288529.42 (206366.14 to 388675.38)	63.45 (45.24 to 85.83)	0.05 (−0.48 to 0.59)	−0.33 (−0.88 to 0.22)
**Oceania**	52.71 (35.98 to 80.45)	1.81 (1.22 to 2.75)	1518.34 (1044.35 to 2259.42)	44.89 (30.58 to 67.64)	163.54 (105.61 to 251.21)	2.50 (1.61 to 3.85)	4597.42 (2973.86 to 6977.80)	59.44 (38.53 to 91.12)	1.36 (1.26 to 1.46)	1.22 (1.12 to 1.32)
**South Asia**	5746.76 (4262.95 to 7581.72)	1.03 (0.75 to 1.40)	168076.88 (124495.17 to 216529.88)	26.21 (19.55 to 34.49)	18705.79 (13926.78 to 24082.93)	1.36 (1.01 to 1.77)	494121.78 (364436.14 to 634816.32)	33.23 (24.74 to 42.71)	0.94 (0.84 to 1.03)	0.81 (0.71 to 0.91)
**Southeast Asia**	4806.82 (3556.57 to 6148.92)	1.80 (1.33 to 2.30)	147500.11 (107900.06 to 187004.01)	49.74 (36.65 to 63.36)	14422.15 (10350.09 to 19194.01)	2.35 (1.69 to 3.11)	400599.72 (287232.68 to 530624.60)	59.59 (42.84 to 79.40)	0.73 (0.62 to 0.84)	0.41 (0.31 to 0.52)
**Southern Latin America**	2085.23 (1623.19 to 2578.43)	4.51 (3.52 to 5.59)	52823.54 (41982.26 to 65341.34)	112.66 (89.60 to 139.48)	3695.77 (2862.89 to 4775.90)	4.40 (3.42 to 5.66)	81211.00 (63664.62 to 103324.59)	98.94 (77.26 to 125.46)	0.29 (0.10 to 0.48)	−0.16(−0.33 to 0.01)
**Southern sub-Saharan Africa**	1379.25 (999.95 to 1916.95)	5.25 (3.78 to 7.31)	35119.59 (25997.71 to 47771.30)	123.39 (90.95 to 169.76)	2374.59 (1793.92 to 2995.29)	4.62 (3.47 to 5.83)	53858.94 (40645.706 to 7981.52)	96.04 (72.62 to 121.52)	−0.72 (−1.28 to −0.16)	−1.16 (−1.78 to −0.54)
**Tropical Latin America**	2551.58 (2067.72 to 3081.35)	2.97 (2.39 to 3.59)	68879.66 (55878.73 to 83851.94)	70.34 (57.05 to 85.40)	6589.14 (5271.72 to7952.07)	2.78 (2.20 to 3.36)	151728.78 (123519.05 to 181417.75)	61.47 (50.00 to 73.34)	0.01 (−0.10 to 0.12)	−0.27 (−0.39 to −0.16)
**Western Europe**	73269.38 (57295.52 to 87910.35)	12.24 (9.63 to 14.71)	1511490.50 (1184705.49 to 1822581.18)	262.76 (205.88 to 316.74)	92291.88 (73466.78 to 110974.05)	9.55 (7.64 to 11.54)	1632548.58 (1304335.12 to1978701.68)	184.94 (147.29 to 225.48)	−0.76 (−0.82 to −0.70)	−1.14 (−1.19 to −1.10)
**Western sub-Saharan Africa**	642.07 (447.05 to 938.08)	0.72 (0.49 to 1.07)	18380.43 (12903.10 to 25635.23)	18.69 (13.03 to 26.71)	1281.63 (907.07 to 1845.78)	0.68 (0.48 to 1.01)	37461.29 (26536.10 to 52501.40)	17.25 (12.19 to 24.62)	−0.25 (−0.35 to −0.14)	−0.33 (−0.42 to −0.24)

Abbreviations: DALY, disability-adjusted life year; EAPC, estimated annual percentage change; UI, uncertainty interval.

### 3.2. Age distribution of global cancer burden attributable to occupational risk factors in 2019

**Figure 2:**
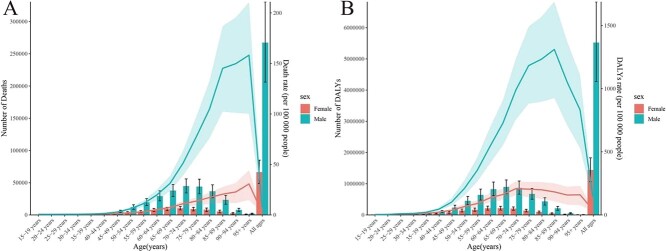
The number and rate of cancers attributable to occupational risks, by sex and age. A, The number of deaths, and age standardized death rate. B, The number of disability-adjusted life years (DALYs), and age-standardized DALY rate.

In 2019, the global death rate and DALY rate of cancer attributable to occupational risk factors increased with age and then decreased. Specifically, the death rate of cancer significantly increased with age, reaching a peak at the age of 85 to 89 years, and then gradually decreased after the age of 90 years. The DALY rate significantly increased with age, reaching a peak at the age of 75 to 79 years, and then significantly decreasing (Tables S1 and S2; Figure 2A and B).

**Figure 3:**
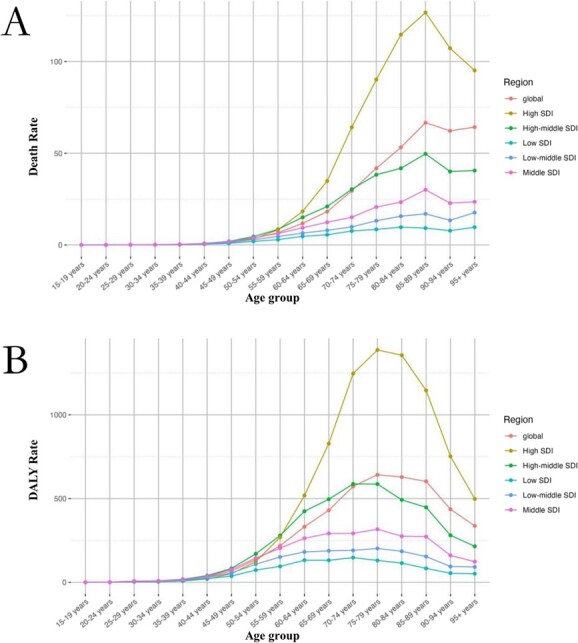
The rates of cancers attributed to occupational risks from 1990 to 2019, by region and age. A, Death rate. B, Disability-adjusted life year (DALY) rate.

Furthermore, in 2019, regarding the age-related global distribution of cancer burden attributable to occupational risk factors, the trends in men and in women, respectively, were similar to the overall trend. Moreover, the age-related trends in death rates and DALY rates of cancer were largely consistent across regions with different SDI levels, with both rates increasing significantly with age. Specifically, the death rate in regions with different SDI levels reached a peak at the age of 85-89 years, and the DALY rate reached a peak at the age of almost at 75–79 years; these results are mostly consistent with the corresponding global trends by age (Tables S1 and S2; Figure 3A and B).

**Figure 4:**
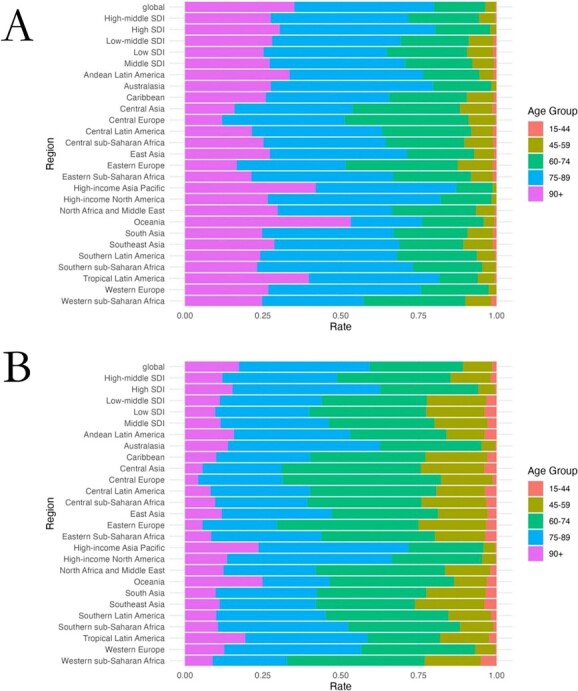
The death and disability-adjusted life year (DALY) rates of cancer attributed to occupational risks in different age groups. A, Death rate in 2019. B, DALY rate in 2019.

In terms of the age distribution of the global cancer burden attributable to occupational risk factors in various regions, the death rates and DALY rates of people aged over 90 years in high-income Asia-Pacific, Oceania, and tropical Latin America accounted for a relatively high proportion relative to the rates for all age groups, and were higher than the global average. In contrast, the death rates and DALY rates of people aged over 90 years in Central Asia, Central Europe, and Eastern Europe were lower than the global average (Tables S1 and S2; Figure 4A and B).

### 3.3. Gender distribution of global cancer burden attributable to occupational risk factors from 1990 to 2019

From 1990 to 2019, the numbers of cancer deaths and DALYs in men and women attributable to occupational risk factors increased (Table 1). However, during this period, the ASDR and age-standardized DALY rate of cancer in men attributable to occupational risk factors decreased. Specifically, the former decreased from 10.08 to 7.54, and the corresponding EAPC was −0.90 (95% CI: −0.99 to −0.82); the latter decreased from 206.29 to 144.38, and the corresponding EAPC was −1.20 (95% CI: −1.26 to −1.13) in men; both cases indicated an overall downward trend. Similarly, the age-standardized DALY rate decreased from 35.24 to 33.10 in women, and the corresponding EAPC was −0.33 (95% CI: −0.38 to −0.27), indicating an overall downward trend.

Overall, from 1990 to 2019, although the ASDR and age-standardized DALY rate of cancer decreased faster in men than in women, the numbers of cancer deaths and DALYs, ASDRs for cancer, and age-standardized DALY rates of cancer were higher in men than in women (Table 1).

### 3.4. Distribution of cancer burden attributable to occupational risk factors in various regions and countries from 1990 to 2019

In 2019, among the 21 geographical regions worldwide, the 3 regions with the highest ASDRs for cancer per 100 000 people attributable to occupational risk factors were high-income Oceania (9.97), Western Europe (9.55), and high-income North America (7.99), whereas the 3 regions with the lowest ASDRs for cancer per 100 000 people attributable to occupational risk factors were South Asia (1.36), eastern sub-Saharan Africa (1.18), and western sub-Saharan Africa (0.68). From 1990 to 2019, East Asia (EAPC = 1.21) exhibited the fastest increase in ASDR for cancer attributable to occupational risk factors, followed by Oceania (1.36) and Central Europe (1.50), whereas Andean Latin America (−2.11), Eastern Europe (−1.63), and high-income North America (−1.63), exhibited the fastest decreases in ASDRs for cancer attributable to occupational risk factors (Tables 1, S3, and S4).

In 2019, the regions with the highest age-standardized DALY rate attributable to occupational risk factors were high-income Western Europe (184.94), Australasia (181.71), and high-income North America (141.32), whereas those with the lowest age-standardized DALY rate attributable to occupational risk factors were western sub-Saharan Africa (17.25), eastern sub-Saharan Africa (27.96), and South Asia (33.23). From 1990 to 2019, the annual standardization rate of DALYs attributable to occupational risk factors increased the most in Oceania (1.22), Central Europe (0.90), and South Asia (0.81), whereas it decreased the most in high-income North America (−2.27), Andean Latin America (−2.13), and Eastern Europe (−2.02) (Tables 1, S3, and S4).

In 2019, of 204 countries, Greenland had the highest ASDR for cancer attributable to occupational risk factors (20.23), followed by Monaco (18.26) and the Netherlands (14.96). From 1990 to 2019, Peru had the highest decrease in ASDR for cancer attributable to occupational risk factors (−3.99; 95% CI: −4.57 to −3.42), followed by Singapore (−3.05) and Kazakhstan (−2.98); in contrast, Georgia had the highest increase in ASDR for cancer attributable to occupational risk factors (5.04), followed by Croatia 4.01 (95% CI: 3.04-5.00) and Honduras (3.54) (Tables 1, S5, and S6).

In 2019, of 204 countries, Greenland had the highest age-standardized DALY rate attributable to occupational risk factors (390.00), followed by Monaco (357.37) and Andorra 283.8; in contrast, Nigeria had the lowest annual standardization rate of cancer DALYs attributable to occupational risk factors (11.10), followed by Mali (13.30; 95% CI: 8.18-20.8) and Mauritania (15.12) (Tables 1, S5, and S6).

From 1990 to 2019, the country with the largest decrease in the number of DALYs due to cancer attributable to occupational risk factors was Peru (−3.78), followed by Singapore (−3.46) and Kazakhstan (−3.35) (Tables 1, S5, and S6;Figure S1).

### 3.5. Relationship between the global burden of cancer attributable to occupational risk factors and SDI levels from 1990 to 2019

According to Table 1, in 1990 and 2019 in high-SDI countries, the numbers of deaths, DALYs, ASDRs, and age-standardized DALY rates due to cancer attributable to occupational risk factors were high. In 1990, the ASDR due to cancer attributable to occupational risk factors in high-SDI regions (10.46) was much higher than that in low-SDI regions (0.98). By 2019, the ASDR due to cancer attributable to occupational risk factors in high-SDI regions was 7.67, which was again higher than that in low-SDI regions (1.17). This shows that as the SDI level of a region increased, its burden of cancer attributable to occupational risk factors also increased (Tables 1 and S4).

However, between 1990 and 2019, the EAPCs of the ASDRs and age-standardized DALY rates of cancer attributable to occupational risk factors in medium-to-high SDI regions were −0.43 and −0.89, respectively, whereas those in high-SDI regions were −1.08 and −1.53, respectively. This indicates that between 1990 and 2019, the ASDRs and DALYs of cancer attributable to occupational risk factors in these regions decreased. In contrast, during the same period, the ASDRs and age-standardized DALY rates in low-, low-medium-, and medium-SDI regions increased (Tables 1 and S4).

Figure S2 shows that as the SDI levels increased, the ASDR and age-standardized DALY rate of cancer attributable to occupational risk factors increased rapidly. Specifically, in countries with an SDI value that was initially less than 0.7, the ASDR and age-standardized DALY rate of cancer attributable to occupational risk factors increased slowly as the SDI value increased. In contrast, in countries with an SDI value that was initially greater than 0.7, the ASDR and age-standardized DALY rate of cancer attributable to occupational risks increased at the same rate at which the SDI value increased. This indicates that the cancer burden of high-SDI regions increased significantly (Tables 1 and S4; Figure S2).

### 3.6. Factors influencing global cancer EAPCs attributable to occupational risk factors

The correlation analysis revealed a significant correlation between EAPC and ASDR (1990) and between EAPC and age-standardized DALY rate (1990) for cancer attributable to occupational risk factors and for SDI values (2019). In particular, there was a significant and negative correlation between EAPC and ASDR (1990; *P* < .05) and age-standardized DALY rate (1990, *P* < .05) for cancer attributable to occupational risk factors (Figure S3A and B).

There was a negative correlation between EAPC and SDI in various countries in 2019 (*P* value <.05). When the SDI value of a country was initially no greater than 0.5, there was a positive correlation between EAPC and SDI; this indicates that from 1990 to 2019, low-SDI countries exhibited a rapid increase in the incidence of cancer attributable to occupational risk factors as their SDI values increased. In contrast, when the SDI value of a country was initially 0.5 to 1.0, there was a significant and negative correlation between EAPC and SDI; this indicates that from 1990 to 2019, high-SDI countries exhibited a decrease in the incidence of cancer as their SDI values increased (Figure S3C).

## 4. Discussion

This study was based on the latest research on the global cancer burden attributable to occupational risk factors, as determined from data reported in GBD 2019. Our results show that the spatial distribution of this burden varied substantially across countries and regions.^8–10^ From 1990 to 2019, the age-standardized rate of cancer burden attributable to occupational risk factors increased. This suggests that this cancer burden is constantly growing, which may be due to the general increase in the level of occupational risk factors worldwide.^19^ From 1990 to 2019, the number of cancer deaths and the total number of DALYs due to cancer increased, largely due to the growth and aging of the world population. Moreover, the absolute number of cancer cases continued to grow, especially in developing countries.^19–21^

However, from 1990 to 2019, the ASDR and age-standardized DALY rate of cancer attributable to occupational risk factors decreased in medium-, medium-high-, and high-SDI regions. This may be due to changes in people’s health awareness and behavior in such regions; that is, compared with people in lower-SDI regions, people in higher-SDI regions usually have a greater awareness of their health, and thus may be more willing to reduce environmental risks to health, which may help reduce the risk of certain types of cancer. Furthermore, compared with low-SDI regions, high-SDI regions often have more advanced health care systems, such as better disease-screening and early diagnosis facilities, and more effective treatment services, which may lead to a decrease in the ASDR and DALY rate for cancer.^8^ Moreover, compared with people in low-SDI regions, those in high-SDI regions usually have more resources and opportunities to maintain their health, including an appropriate diet, regular physical examinations, and sufficient rest and exercise, which reduce the harm caused by occupational cancer risk factors.^22^^,^^23^

In terms of gender distribution, the burden of cancer attributable to occupational risk factors was higher in men than in women. This may be because men are more likely than women to work in environments with high occupational risk factors for cancer. Men have a higher prevalence of exposure to other risk factors for cancer compared to women. Unhealthy habits such as smoking, being overweight, consuming alcohol, and eating meat are more commonly found in men.^24^ A study on French workers revealed that male clerks and manual workers are at a higher risk of developing smoking- and alcohol-related cancers, a trend not observed among women.^25^

Numerous studies have shown that high levels of occupational risks are strongly associated with an increased risk of bladder, breast, colon, endometrial, kidney, and gastric cancer, and esophageal adenocarcinoma. However, there were differences in this trend between regions and countries, which may be due to differences between their socioeconomic levels and in their populations’ level of awareness of and concern about occupational risk factors for cancer.^23^^,^^26^

In terms of age distribution, the cancer burden attributable to occupational risk factors was the highest among the elderly. This may be because people’s physical function level and immunity usually decline with age, which increases the risk of cancer and other diseases. Moreover, older adults have high baseline cancer incidence and death rates.^27^ Therefore, encouraging the elderly to carry out occupational cancer-risk prevention may help to reduce their cancer burden. However, it was found by other researchers that compared with the survival rate of cancer in a non-elderly population, the survival rate in an elderly population was higher, resulting in a corresponding increase in their cancer burden.^28^ Moreover, that study found that cancer survivors are usually older than those who have never had cancer. Therefore, the cancer burden in the elderly population that is attributable to occupational risk factors may also be due to this population containing a high proportion of cancer survivors.^29^ However, this does not mean that the cancer burden in non-older adults that is attributable to occupational risk factors can be ignored, because these people are exposed to a higher level of such risks than older adults.^30^^,^^31^ Additionally, certain cancers resulting from occupational hazards, like asbestos-induced lung cancer, have long latency periods. Research suggests that asbestos-induced cancer deaths may continue for 40-50 years,^32^ emphasizing the importance of regular medical check-ups for workers previously exposed to asbestos even after leaving the work environment.

The regional and national distributions of the burden of cancer attributable to occupational risk factors have increased rapidly in developing countries and low-income countries. There are 4 possible explanations for this phenomenon. First, work environments and safety regulations in high-SDI countries or regions are usually strict, which helps to reduce exposure to occupational risk factors for cancer. Conversely, in low-SDI countries or regions, working environments may be poor, and safety regulations may be insufficient or not fully implemented, thereby increasing exposure to occupational risk factors for cancer. Besides, in low-SDI countries or regions, people’s health awareness and education levels may be low, and thus they may not be sufficiently aware of occupational health risks and are unlikely to take steps to avoid or reduce them.

We also found that the global cancer burden was related to levels of socioeconomic development; in particular, SDI values, which reflect socioeconomic status, were related to cancer burdens attributable to exposure to occupational risk factors for cancer. Specifically, the global burden of cancer increased overall; however, the burden was highest in high-SDI regions but decreased over time, whereas the burden was lowest in low-SDI regions but increased over time.^4^^,^^5^^,^^33^ High-SDI regions had higher burden of cancer than lower SDI regions in the past, reflecting that industrialization and cumulative occupational exposures that occurred decades ago in the high SDI regions.^22^

The increase in the burden of cancer in low-SDI regions may be related to their harsh environments, high levels of occupational risk factors for cancer, and their absence of effective diagnosis and treatment services.^34^^,^^35^ The lower burden in low-SDI regions than in high-SDI regions may be caused by the high levels of occupational risk factors for cancer in the former regions being offset by population growth and aging in some countries. Therefore, demographic trends will continue to result in reduced cancer burdens in high-SDI regions.^30^^,^^32^ The burden of cancer attributable to occupational risk factors might have remained high in high-SDI regions from 1990 to 2019 due to the high detection rates and high reporting rates for cancer in these regions.^4^^,^^5^^,^^33^^,^^35–39^

We found that occupational risk factors for cancer had a strong effect on the EAPC of the global ASDR for cancer between 1990 and 2019. This may be due to increasing exposure to occupational risk factors due to the development of society; that is, people’s lifestyles have undergone significant changes, and the exposure to and intensity of occupational risk factors for cancer have increased. In addition, with the development of science and technology, people may be increasingly exposed to carcinogens (such as chemicals, radiation, and dust) in some working environments. Therefore, preventing occupational risk factors and changing unhealthy lifestyles are important means of preventing cancer and decreasing cancer burdens.^35^

In brief, geographical differences exist in the distribution and trend of cancer burdens attributable to occupational risk factors. In some countries with certain levels of socio-economic development, such as Georgia, Honduras, and Croatia, the cancer burden has increased rapidly. This may be related to an increase in the level of occupational risk factors for cancer in these countries.^11^^,^^40^^,^^41^ Additionally, although the global age-standardized burden of cancer attributable to occupational risk factors declined from 1990 to 2019, the burden is higher in men than in women, and higher in middle-aged and older adults than in younger people.^9^^,^^10^

Cancer burdens have increased as regions’ SDI values have increased, especially in high-SDI regions. This finding will aid the development of targeted strategies for cancer prevention and occupational risk-factor management in different regions. Similarly, we found that as baseline cancer burdens increased, the rate of increase in ASDRs for cancer slowed. Thus, to effectively prevent and control cancer, attention must be paid to regions and populations with high cancer burdens and rapidly growing cancer burdens.^6^^,^^41^

The findings of this study make several novel contributions to the literature. First, the impacts of occupational risk factors on the global cancer burden were determined. This global-level research enhances our understanding of and quantifies the contribution of risk factors to global cancer burdens, and provides an important basis for formulating public health strategies and interventions. Second, this study revealed the change trends in global cancer age-standardized rates attributable to exposure to occupational risk factors, and examined other influencing factors, such as gender and SDI values.

However, this study has some limitations. First, the study relied on GBD 2019 data, which may be inaccurate or incomplete for some regions or countries, and thus our results may contain bias. Also, this study focuses on the global overall incidence of occupational cancer, and there is a lack of research on specific occupational risk factors. Second, this study focused on the global situation, and thus might have failed to capture the situation in certain regions or countries in detail. Third, this study examined the impact of occupational risk factors on the global cancer burden and thus might not have captured the impact of other risk factors. In addition, global-level research may not fully consider regional differences in, for example, risks, population structures, and socioeconomic conditions, which can affect results.

Ongoing globalization and industrialization may cause the number of occupational risk factors for cancer to increase, so more research and efforts are needed to control these risks. In developing countries and low-income countries, resources and efforts must be increased to improve work environments and safety regulations to reduce the impact of occupational risk factors for cancer. In developed and high-income countries, although the burden of cancer attributable to occupational risk factors has decreased, the large baseline burden means that increased attention should be paid to the prevention and control of these factors.

For example, taking corresponding measures for occupational carcinogenic factors, conducting risk assessments of carcinogenic factors, and controlling their risk to the lowest level. For example, for workers who work under sunlight for long periods of time, quantifying cumulative exposure to solar ultraviolet radiation can accurately estimate the risk of occupational skin cancer. A new method proposed by Paulo et al^42^ uses a personal electronic dosimeter integrated with a digital platform to evaluate occupational solar ultraviolet radiation dose. In addition, qualified protective measures are also a key factor in protecting the health of workers, such as wearing qualified masks, protective clothing, etc. Last, establish a sound health monitoring system, conduct regular physical examinations for workers, screen for highly sensitive indicators of cancer, and prevent cancer early. At the same time, strengthen publicity and education for workers to enhance occupational health awareness.

Overall, this study provides a new perspective on the change trends of global cancer burdens and reveals some important factors affecting these change trends. Therefore, our findings enhance our understanding of the drivers of changes in cancer burdens and will support the formulation of effective cancer prevention and control strategies. However, our findings also reveal some areas that require further research, such as the specific occupation-related factors causing changes in cancer burdens, and how these factors can be managed to effectively prevent and control cancer.

## Supplementary Material

Web_Material_uiae040

## Data Availability

The datasets generated and analyzed during the current study are available from the corresponding author on reasonable request.
