# Supplementary material for: Global, regional, and national burden of cancers attributable to occupational risks from 1990 to 2019
Source: J Occup Health. 2024 Jul 24;66(1):uiae040. doi: 10.1093/joccuh/uiae040 (PMC11378634; doi:10.1093/joccuh/uiae040)
Supplement: Web_Material_uiae040 [file web_material_uiae040.docx]

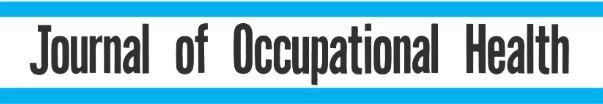


***Journal of Occupational Health***

# Change of Authorship Form

(Must be Completed and Signed by ALL Authors)

# Journal of Occupational Health’s Policy

Authorship is limited to those who have made a significant contribution to the design and execution of the work described. Any contributors whose participation does not meet the criteria for authorship should be acknowledged but not listed as an author. For a detailed definition of authorship, please see the [International Committee of](https://www.icmje.org/recommendations/browse/roles-and-responsibilities/defining-the-role-of-authors-and-contributors.html) [Medical Journal Editors (ICMJE) definitions of authors and contributors.](https://www.icmje.org/recommendations/browse/roles-and-responsibilities/defining-the-role-of-authors-and-contributors.html)

The Journal does not allow ghost authorship, where an unnamed author prepares the article with no credit, or guest/gift authorship, where an author who made little or no contribution is listed as an author. The Journal follows Committee on Publication Ethics (COPE) guidance on investigating and resolving these cases. For more information, please see the [OUP Publication Ethics page.](https://academic.oup.com/pages/authoring/journals/preparing_your_manuscript/ethics#Authorship)

Natural language processing tools driven by artificial intelligence (AI) do not qualify as authors, and the Journal will screen for them in author lists. The use of AI (for example, to help generate content or images, write code, process data, or for translation) should be disclosed both in cover letters to editors and in the Methods or Acknowledgements section of manuscripts. Please see the [COPE position statement on Authorship and AI](https://publicationethics.org/cope-position-statements/ai-author) for more details.

After manuscript submission, no authorship changes (including the authorship list, author order, and who is designated as the corresponding author) should be made without the approval of the editor. All co-authors must agree on the change(s), and neither the Journal nor the publisher mediates authorship disputes. If individuals cannot agree on the authorship of a submitted manuscript, contact the editorial office. The dispute must be resolved among the individuals and their institution(s) before the manuscript can be accepted for publication. If an authorship dispute or change arises after a paper is accepted, contact OUP’s Author Support team. COPE provides [guidance for authors on resolving authorship disputes.](https://publicationethics.org/resources/guidelines-new/how-handle-authorship-disputesa-guide-new-researchers)

# Please fill out the Form below and send to [joh.editorialoffice@oup.com.](mailto:joh.editorialoffice@oup.com)

1. Manuscript ID: JOH-2023-0547-OA.R1

Manuscript Title: Global, regional, and national burden of cancers attributable to occupational risks from 1990 to 2019

1. **Reasons for Authorship Change**: Please provide a detailed explanation of the reason for the requested authorship change in the space below. We reserve the right to request evidence of authorship, and changes to authorship will be made at the discretion of the Editors. Authors are encouraged to provide any additional materials to support the request in the initial application.

Because the corresponding author has already resigned. Zhou Lihong changed her job in March. She was in Shanghai Shuguang Hospital before and was transferred to Shanghai Pudong New Area Hospital of Traditional Chinese Medicine after March.

Page 1 of 5

Description of the change: (new author(s) have been added; change in the order of authorship; An author wishes to remove his/her name)

1. **Author agreement**: The corresponding author should act as a main point of contact and provide details of authorship and author’s contributions, although these duties may be delegated to one or more co-authors. Editorial office reserve the right to request signed statement of agreement for the requested change from all listed authors and from the author to be removed or added.

We require consent from all the authors (including from the added/removed co-author) confirming that they are satisfied with the change. Ideally, this will be in the form of an email, preferably from the institution address of the relevant authors

# Original Authorship

LIST ALL AUTHORS in the same order as the original (first) submission. Use an extra sheet if there are more than 10 authors.

| Please indicate corresponding author(s) by adding asterisk | Name | Affiliation |
| --- | --- | --- |
| author (1) | Shiliang Ling | Department of Oncology, Ningbo Hospital of Traditional Chinese Medicine, Ningbo, Zhejiang, China |
| author (2) | Lihong Zhou***** | Department of Medical Oncology & Cancer Institute of Integrative Medicine, Shuguang Hospital, Shanghai University of Traditional Chinese Medicine, Shanghai, China |
| author (3) | Yanfeng Wu | Department of Anesthesiology, Ningbo Hospital of Traditional Chinese Medicine, Ningbo, Zhejiang, China |
| author (4) | Xiaoling Zhang | Department of Oncology, Ningbo Hospital of Traditional Chinese Medicine, Ningbo, Zhejiang, China |
| author (6) | Wulong Han | Department of Oncology, Ningbo Hospital of Traditional Chinese Medicine, Ningbo, Zhejiang, China |
| author (7) | Lihua Cui***** | Department of Oncology, Ningbo Hospital of Traditional Chinese Medicine, Ningbo, Zhejiang, China |
| author (8) | Zhiyu Luan | Department of Oncology, Ningbo Hospital of Traditional Chinese Medicine, Ningbo, Zhejiang, China |
| author (9) |  |  |
| author (10) |  |  |

# New Authorship

All authors must sign in the list below agreeing to the new changes in authorship. The authorship order and appointed corresponding authors must match the new title page of the manuscript. Signatures below certify


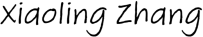

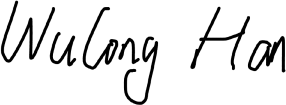

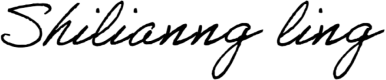

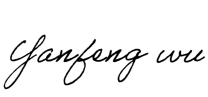
compliance with the author responsibilities on the next page. List ALL AUTHORS in the same order as the new version.

| Please indicate corresponding author(s) by adding asterisk | Title  (Mr./Ms./Mrs.  /Dr./Prof.) | Name | Email | Affiliation | Signature&Dat e |  |
| --- | --- | --- | --- | --- | --- | --- |
| author (1) | Dr. | Shiliang Ling | Sliang621@ 163.com | Department of Oncology, Ningbo Municipal Hospital of Traditional Chinese Medicine(TCM), Affiliated Hospital of Zhejiang Chinese Medical University, Ningbo,  Zhejiang, China |  |  |
| author (2) | Dr. | Lihong Zhou | zlhtcm@ho tmail.com | Department of Spleen and Stomach, Shanghai Pudong New Area Traditional Chinese Medicine Hospital, Shanghai, China | 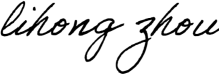 |  |
| author (3) | Dr. | Yanfeng Wu | 153271885  @qq.com | Department of  Anesthesiology, Ningbo Municipal Hospital of Traditional Chinese Medicine(TCM), Affiliated Hospital of Zhejiang Chinese Medical University, Ningbo,  Zhejiang, China |  |  |
| author (4) | Dr. | Xiaoling Zhang***** | nbzhangxl @tom.com | Department of Oncology, Ningbo Municipal Hospital of Traditional Chinese Medicine(TCM), Affiliated Hospital of Zhejiang Chinese Medical University, Ningbo,  Zhejiang, China |  |  |
| author (5) | Dr. | Wulong Han | hanwulong 650213@16  3.com | Department of Oncology, Ningbo Municipal Hospital of Traditional Chinese Medicine(TCM), Affiliated Hospital of Zhejiang Chinese Medical University, Ningbo,  Zhejiang, China |  |  |

| author (6) | Dr. | Lihua Cui | c13957845 [405@163.c](mailto:405@163.c)  om | Department of Oncology, Ningbo Municipal Hospital of Traditional Chinese Medicine(TCM), Affiliated Hospital of Zhejiang Chinese Medical University, Ningbo,  Zhejiang, China |  |
| --- | --- | --- | --- | --- | --- |
| author (7) | Dr. | Zhiyu Luan | 278659192  @qq.com | Department of Oncology, Ningbo Municipal Hospital of Traditional Chinese Medicine(TCM), Affiliated Hospital of Zhejiang Chinese Medical University, Ningbo,  Zhejiang, China |  |


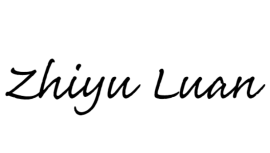

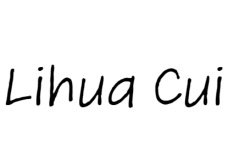


# Authors to be removed (If any)

|  | name | affiliation | Signature&Date |
| --- | --- | --- | --- |
| author (1) |  |  |  |
| author (2) |  |  |  |
| author (3) |  |  |  |
| author (4) |  |  |  |
| author (5) |  |  |  |

1. **Authors’ contributions:**

Journal of Occupational Health follows the International Committee of Medical Journal Editors ([ICMJE)](http://www.icmje.org/recommendations/browse/roles-and-responsibilities/defining-the-role-of-authors-and-contributors.html) guidelines on defining the role of authors and contributors. Authorship is limited to those who have made a significant contribution to the design and execution of the work described. Though not mandatory, [CRediT](https://credit.niso.org/) author statement (shown on list below) helps to clarify the contributions of the authors.

Please list all the author’s Contribution here:

| Author’s Contribution | |
| --- | --- |
| Shiliang Ling | Conceptualization and Writing - original draft |
| Lihong Zhou | Conceptualization and Writing - original draft |
| Yanfeng Wu | Conceptualization and Writing - original draft |
| Xiaoling Zhang | Data curation and Formal analysis |
| Wulong Han | Data curation and Formal analysis |
| Lihua Cui | Funding acquisition and Writing - review & editing |
| Zhiyu Luan | Data curation, Formal analysis, Funding acquisition and Writing - review & editing |
| author (8) |  |
| author (9) |  |
| author (10) |  |

Contributor Roles Taxonomy (CRediT)

| Conceptualization | Ideas; formulation or evolution of overarching research goals and aims. |
| --- | --- |

| Data curation | Management activities to annotate (produce metadata), scrub data and maintain research data (including software code, where it is necessary for interpreting the data itself) for initial use and later re-use. |
| --- | --- |
| Formal analysis | Application of statistical, mathematical, computational, or other formal techniques to analyze or synthesize study data. |
| Funding acquisition | Acquisition of the financial support for the project leading to this publication. |
| Investigation | Conducting a research and investigation process, specifically performing the experiments, or data/evidence collection. |
| Methodology | Development or design of methodology; creation of models. |
| Project administration | Management and coordination responsibility for the research activity planning and execution. |
| Resources | Provision of study materials, reagents, materials, patients, laboratory samples, animals, instrumentation, computing resources, or other analysis tools. |
| Software | Programming, software development; designing computer programs; implementation of the computer code and supporting algorithms; testing of existing code components. |
| Supervision | Oversight and leadership responsibility for the research activity planning and execution, including mentorship external to the core team. |
| Validation | Verification, whether as a part of the activity or separate, of the overall replication/reproducibility of results/experiments and other research outputs. |
| Visualization | Preparation, creation and/or presentation of the published work, specifically visualization/data presentation. |
| Writing - original draft | Preparation, creation and/or presentation of the published work, specifically writing the initial draft (including substantive translation). |
| Writing - review & editing | Preparation, creation and/or presentation of the published work by those from the original research group, specifically critical review, commentary or revision – including pre- or post-publication stages. |
